# Two-dimensional silicon and carbon monochalcogenides with the structure of phosphorene

**DOI:** 10.3762/bjnano.8.135

**Published:** 2017-06-29

**Authors:** Dario Rocca, Ali Abboud, Ganapathy Vaitheeswaran, Sébastien Lebègue

**Affiliations:** 1 Université de Lorraine, CRM2, UMR 7036, Vandoeuvre-lès-Nancy, F-54506, France; 2 CNRS, CRM2, UMR 7036, Vandoeuvre-lès-Nancy, F-54506, France; 3 Advanced Centre of Research in High Energy Materials (ACRHEM), University of Hyderabad, Prof. C. R. Rao Road, Gachibowli, Hyderabad - 500 046, India

**Keywords:** electronic structure, phosphorene, two-dimensional materials

## Abstract

Phosphorene has recently attracted significant interest for applications in electronics and optoelectronics. Inspired by this material an ab initio study was carried out on new two-dimensional binary materials with a structure analogous to phosphorene. Specifically, carbon and silicon monochalcogenides have been considered. After structural optimization, a series of binary compounds were found to be dynamically stable in a phosphorene-like geometry: CS, CSe, CTe, SiO, SiS, SiSe, and SiTe. The electronic properties of these monolayers were determined using density functional theory. By using accurate hybrid functionals it was found that these materials are semiconductors and span a broad range of bandgap values and types. Similarly to phosphorene, the computed effective masses point to a strong in-plane anisotropy of carrier mobilities. The variety of electronic properties carried by these compounds have the potential to broaden the technological applicability of two-dimensional materials.

## Introduction

Over the last ten years, the interest in two-dimensional materials has increased exponentially [1]. Following the initial report of the existence of graphene [2], it was shown that isolated sheets of other layered compounds, such as h-BN or MoS_2_ among others, could be obtained as well [3–4]. These compounds demonstrate properties of the electronic structure that are markedly different from those of graphene, with, for instance, the existence of a finite bandgap in the band structure [5].

One of the latest newcomers in the family of two-dimensional materials is phosphorene [6–9], which corresponds to a single layer of black phosphorus [10–11], one of the many phases of crystalline phosphorus. Among other properties [12–15], the values of the carrier mobility and of the on–off ratio of transistors made from phosphorene are intermediate between the values of graphene and those of transition metal dichalcogenides, making phosphorene very promising for certain applications. Additionaly, phosphorene is characterized by a strong anisotropy in the carrier mobility [14] and ferroelasticity [16]. Recently, some parent compounds to phosphorene have been considered. For example, P–As compounds were studied theoretically and experimentally [17–18]. Also, arsenene and antimonene [19], SiS [20], and SnS, SnSe, GeS, and GeSe [21–22] compounds with a crystal structure similar to the one of phosphorene were investigated by ab initio calculations. The reliability of ab initio calculations to predict and characterize new two dimensional compounds is now well established. For instance borophene and graphane were predicted theoretically [23–24] before being obtained experimentally [25–26]. In the same way, planar tetracoordinate carbon was predicted computationally and then realized experimentally [27].

In this paper, by employing ab initio methods, we discuss the properties of a number of compounds that can be obtained by chemical replacement in the crystal structure of phosphorene: CS, CSe, CTe, SiO, SiS, SiSe, and SiTe. Differently from phosphorene, no layered bulk structure is known for these compounds. In a recent study Kamal et al. showed that carbon and silicon monochalcogenides are energetically stable and have their lowest energy in a phosphorene-like structure (for SiS and SiSe the puckered phosphorene-like and buckled structures are very close in energy and nearly equally probable at room temperature) [28]. By computing phonon dispersion curves, in this work we show that carbon and silicon monochalcogenides are dynamically stable in a puckered structure. Additionally, we compute band structures using the quantitatively accurate HSE06 hybrid functional [29] and we evaluate effective masses, whose anisotropy represents one of the most exotic properties of phosphorene and phosphorene-like materials. The broad range of properties showed by carbon and silicon monochalcogenides might help to extend the technological applications of two-dimensional materials.

## Theoretical Methods

The calculations presented in this paper have been performed using the Quantum Espresso package [30], which is based on a plane-wave basis set and pseudopotentials. The Perdew–Burke–Ernzerhof (PBE) [31] functional has been used to optimize the structure and to compute phonon dispersion spectra. After optimizing the structure with a force threshold of at least 10^−4^ Ry/Bohr the phonon curves have been computed by using density functional perturbation theory [32]. The kinetic-energy cut-off convergence has been carefully tested for each system and a 12 × 12 × 1 *k*-point grid was used (supposing that the *z* direction is perpendicular to the two-dimensional plane). A vacuum of at least 12 Å has been used to separate periodically repeated images.

Since the PBE functional systematically underestimates the electronic gap with respect to experiments, the band structure was obtained using the HSE06 functional [29]. Because of the numerical cost involved in HSE06 calculations electronic eigenvalues have been computed on a 16 × 16 × 1 *k*-point grid; the band structure was then extracted along the high symmetry directions and interpolated with cubic splines (see below in Figure 3).

Spin–orbit coupling often plays an important role in two-dimensional materials. For example, it was shown that SnSe and GeSe phosphorene-like monochalcogenides are characterized by an anisotropic spin splitting of energy bands, leading to potential applications involving directionally dependent spin transport [22]. In [21] it was suggested that in phosphorene-like SnS, SnSe, GeS, and GeSe the spin–orbit coupling does not significantly change the shape of bands but gives rise to a splitting of the order of a few tens of millielectronvolts. Due to the high computational cost involved in spin-orbit coupling calculations with the HSE06 functional, this effect has not been included in our results. While the inclusion of spin–orbit coupling might slightly affect the values of the gaps and introduce a splitting in some bands, we do not expect a change in the overall conclusions of this work.

In the next section we also show results concerning effective masses, of which the traditional definition requires an approximately quadratic dispersion close to band edges. Differently from graphene, this condition is always satisfied for the materials considered in this work. Effective masses calculations require the accurate evaluation of the partial second derivatives of the band structure in two directions. The 16 × 16 × 1 *k*-point grid used in HSE06 calculations is too coarse and effective masses have been evaluated only from the PBE functional. Since effective masses depend on the band structure dispersion rather then on the gap, this approximation is expected to lead to a reasonable accuracy.

## Results and Discussion

### Structural properties

**Figure 1:**
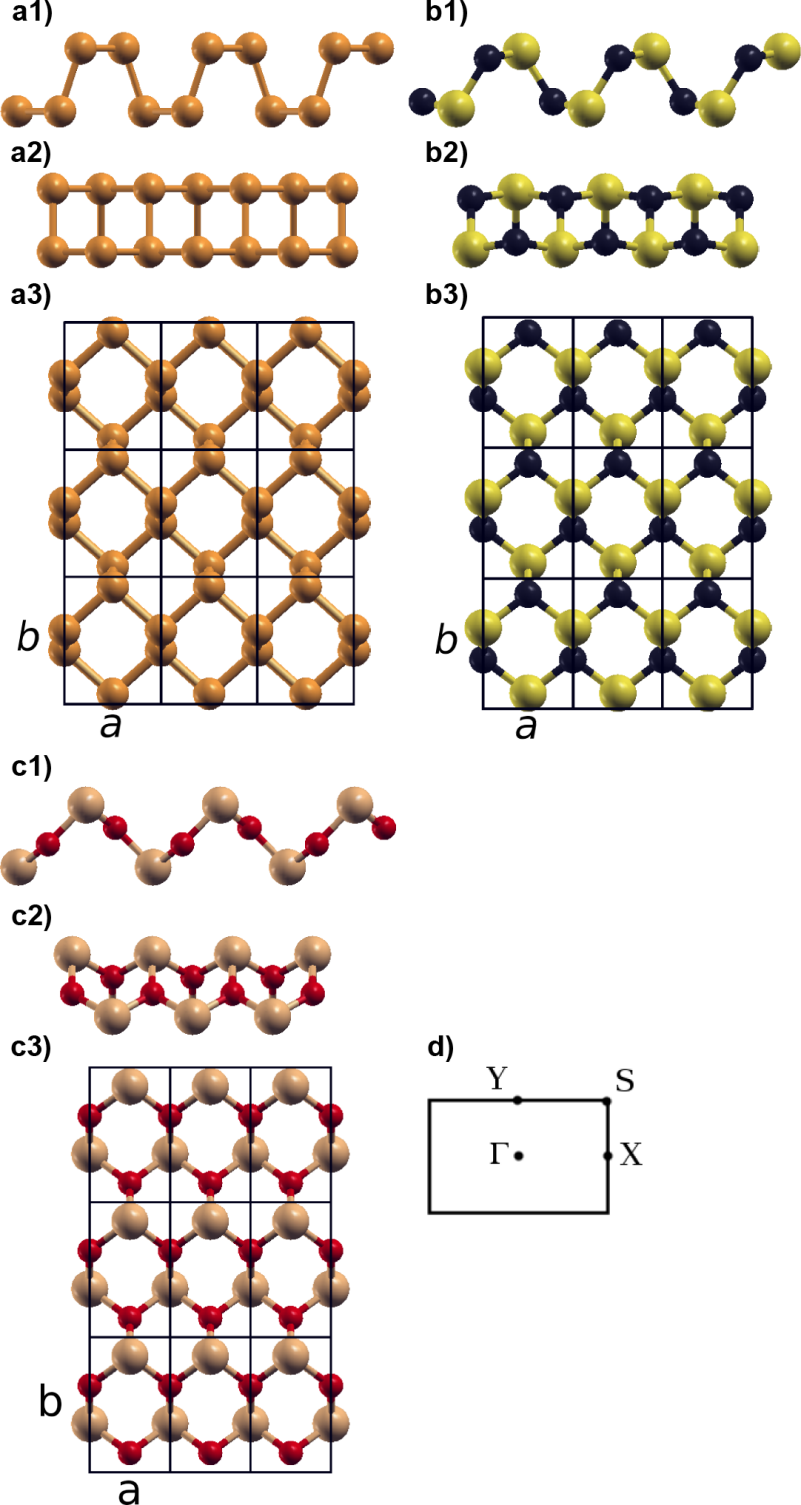
Optimized crystal structures of the following two-dimensional systems: a) phosphorene b) CS, CSe, CTe, SiS, SiSe, and SiTe. c) SiO d) Brillouin zone and high-symmetry points.

In this section we discuss the structural properties and dynamical stability of carbon and silicon-based two-dimensional binary compounds. As a starting point we consider a phosphorene monolayer derived from the experimental structure of black phosphorous [33]. Within this system each phosphorus atom is covalently bonded with three adjacent phosphorus atoms to form a puckered honeycomb structure [33–34] (Figure 1a). For the sake of simplicity this phosphorene monolayer will be denoted as “experimental structure”. Upon optimization at the PBE level of theory the lattice parameters of the experimental structure moderately change from *a* = 3.31330 Å and *b* = 4.374 Å to *a* = 3.298 Å and *b* =4.624 Å, in good agreement with previous calculations [8,35].

In order to obtain the structure of the carbon and silicon monochalcogenides we replaced phosphorus atoms in the experimental structure of phosphorene with carbon (or silicon) and chalcogen atoms (up to tellurium) by preserving the number of valence electrons involved in each bond. This starting geometry was then optimized at the PBE level of theory [36]. We found the CO monolayer to be unstable and to dissociate in independent one-dimensional chains; therefore this system was disregarded in the rest of our study. The geometry obtained for CS is shown in Figure 1b. The main features of the phosphorene structure are kept in this system: Each C atom is bound to three S atoms and vice versa. However, while in phosphorene atoms are distributed on two parallel planes (Figure 1a2), a distortion appears in CS, where C atoms tend to slightly move within the two planes formed by S atoms (Figure 1b2). In the case of CSe, CTe, SiS, SiSe, and SiTe the obtained structures are similar to the monolayer of CS [36]. The case of SiO should be considered separately: As shown in Figure 1c, in this case the distortion is particularly accentuated with respect to the initial geometry. As discussed below, this structure leads to electronic properties rather different from that of phosphorene, in particular concerning effective masses.

To better understand the ionic character of the bonds involved in the new materials we have carried out an analysis of the PBE charge density based on the theory of Bader [37] as implemented by Henkelman et al. [38]. Since silicon has a low electronegativity we found that this atom transfers about 2.4 electrons to O, S, and Se and 0.4 electrons to Te. In carbon monochalcogenides we found that 0.5 electrons were transferred from S to C, 0.8 from Se to C, and 1 from Te to C.

**Figure 2:**
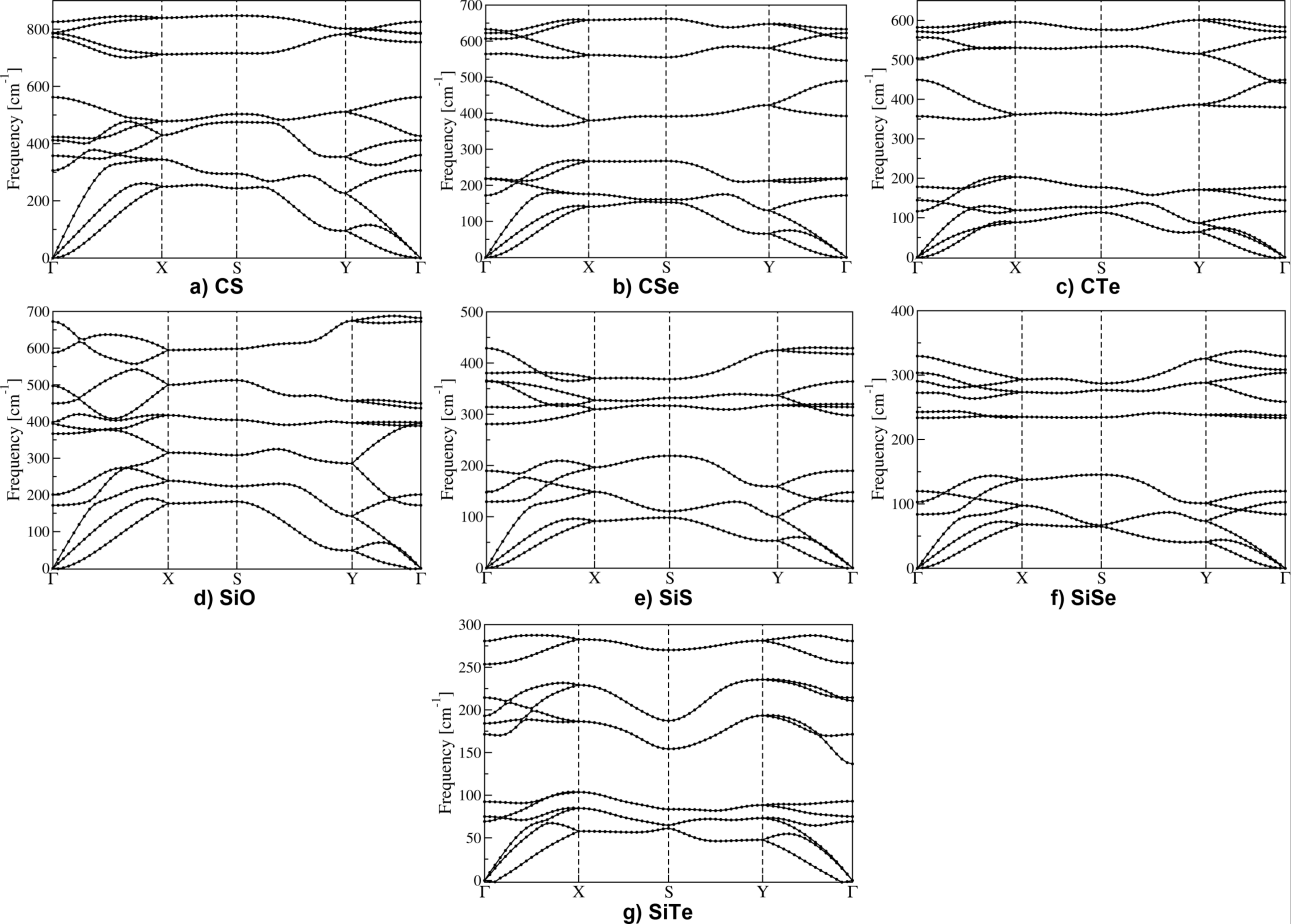
Phonon dispersion curves for the two-dimensional carbon and silicon monochalcogenides introduced in this work: CS, CSe, CTe, SiO, SiS, SiSe, and SiTe.

In order to establish the dynamical stability of these new compounds, we computed phonon dispersion curves by using density functional perturbation theory [32]. Our results in Figure 2 show that no imaginary phonon modes are present and all the compounds considered in this work are dynamically stable (the first Brillouin zone is mapped according to the convention in Figure 1d).

### Electronic properties

**Figure 3:**
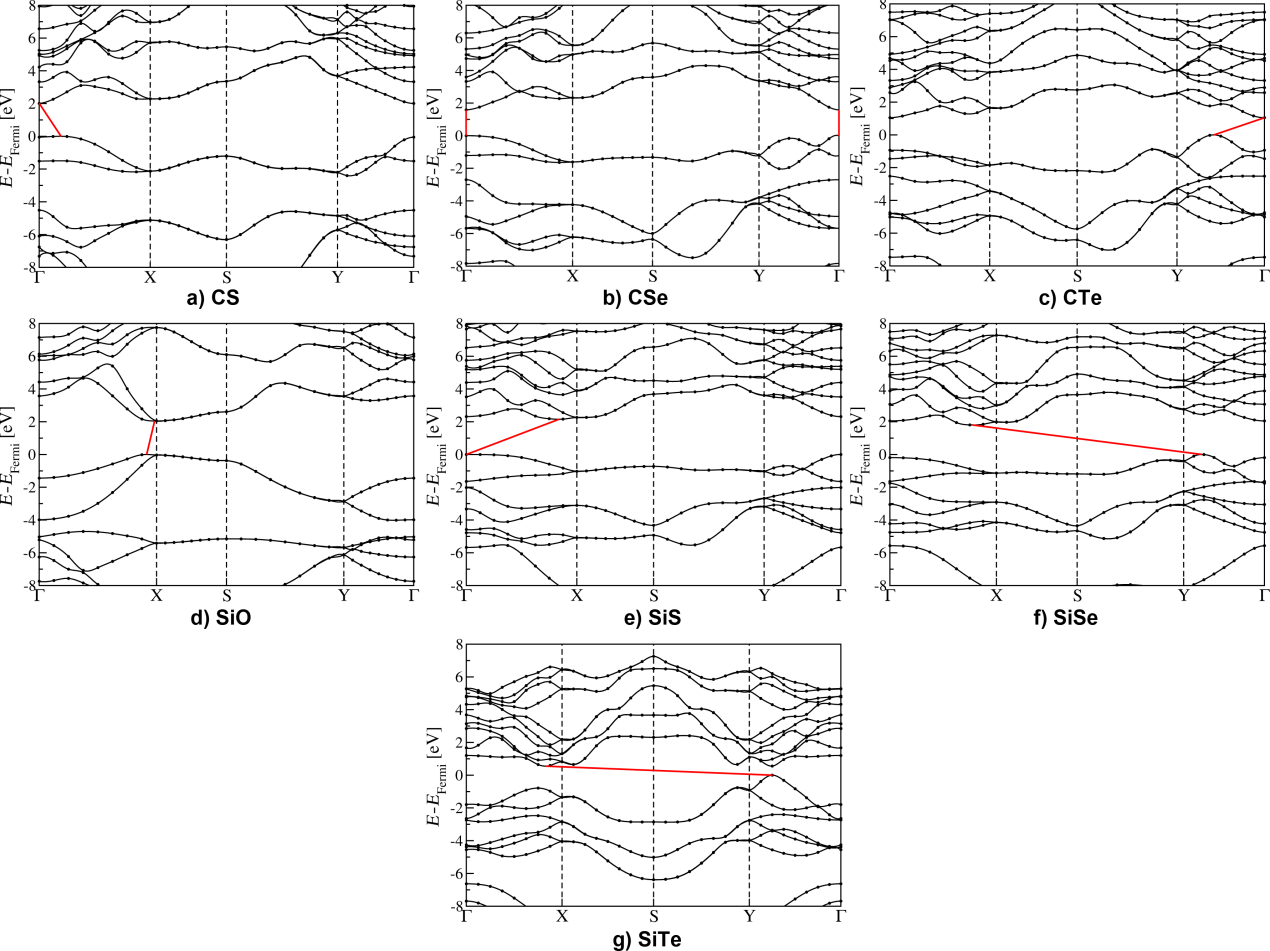
Band structures computed at the HSE level of theory for the two-dimensional carbon and silicon monochalcogenides introduced in this work: CS, CSe, CTe, SiO, SiS, SiSe, and SiTe.

After determining the structural properties we discuss the electronic properties of these phosphorene-like two-dimensional materials, which are fundamental for applications in nanoelectronics and nanophotonics. The band structures computed within the HSE06 approximation [29] are shown in Figure 3 where the gap type is indicated by a red line. The Fermi level has been shifted to zero and the first Brillouin zone is mapped according to the convention in Figure 1d. In Table 1 the values of bandgaps are presented.

**Table 1:** Electronic bandgaps computed at the PBE and HSE level of theory and effective masses for two-dimensional carbon and silicon monochalcogenides introduced in this work. In the table 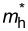 denotes the hole effective mass, 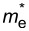 the electron effective mass, and *m*_e_ the electron mass.

material	PBE gap (type)	HSE gap (type)	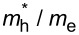	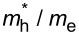	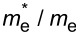	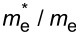
			zigzag direction	armchair direction	zigzag direction	armchair direction

CS	1.13 (indirect)	2.02 (indirect)	1.08	0.22	0.29	0.32
CSe	0.88 (direct)	1.58 (direct)	3.75	0.14	0.35	0.16
CTe	0.56 (indirect)	1.07 (indirect)	0.87	0.20	0.55	0.35
SiO	1.37 (indirect)	2.06 (indirect)	0.36	1.66	0.20	1.60
SiS	1.42 (indirect)	2.16 (indirect)	27.1	0.28	1.10	0.53
SiSe	1.19 (indirect)	1.81 (indirect)	0.87	0.17	0.58	0.21
SiTe	0.39 (indirect)	0.55 (indirect)	0.16	0.06	0.22	0.08
phosphorene	0.91 (indirect)	1.58 (direct)	8.13	0.13	1.24	0.14

Before discussing the characteristics of the two-dimensional carbon and silicon monochalcogenides, some preliminary remarks on phosphorene are necessary. The band gap of phosphorene has been evaluated by different ab initio methods to be about 0.8 or 0.91 eV at the PBE level of theory [35,39], 1.61 eV at the HSE level of theory [35], and 2.0 eV at the GW level of theory [39]. In [8] a value of 1.0 eV was found for the HSE gap, which does not significantly differ from the PBE estimate. As shown in Table 1 the DFT values we found are in good agreement with the results in the literature [35,39]. As previously noted [39], the top of the valence band is slightly shifted from Γ and, accordingly, phosphorene has an indirect gap at the PBE level of theory. However, the gap and the direct gap in Γ differs by less than 10 meV and for this reason phosphorene is commonly considered a direct bandgap semiconductor [8,35]. In Table 1 the HSE gap is denoted as direct. Very likely the difference with respect PBE is due to the coarser *k*-point grid used to sample the Brillouin zone.

The electronic gaps of the phosphorene-like binary compounds have been summarized in Table 1. For the sake of completeness, both PBE and HSE results have been provided but the HSE values have to be considered as more quantitative estimates in comparison to experiments. All the monolayers considered here are semiconducting and the gaps obtained span a wide energy range going from 0.55 eV for SiTe to 2.16 eV for SiS (HSE data). Accordingly, these compounds might contribute to extend the applicability of two dimensional materials to solar cells [40] and photodetection devices [14]. For several technological applications in optoelectronics the discovery of new two-dimensional materials characterized by a direct bandgap is also important. While strictly speaking, only the CSe monolayer has a direct gap, most of the other compounds have a direct bandgap only slightly larger than the minimum energy bandgap: 0.04 eV for CS, 0.02 eV for SiO, and below 0.01 eV for SiTe. Similarly to phosphorene, we can approximately consider these compounds as direct bandgap materials.

An important property of phosphorene is the high mobility of charge carriers and its in-plane anisotropy [14]. Indeed, significantly higher carrier mobilities are found in the armchair direction (along the direction of the lattice constant *b*). This behavior can be qualitatively understood from the band structure by computing effective masses. In agreement with previous calculations [35,41], the computed effective masses of phosphorene in the armchair direction are significantly smaller than in the zigzag direction. Most of the materials studied in this work present a similar behavior (the exceptions being SiO, which has a structure that significantly deviates from phosphorene, and CS, which has similar electron effective masses in the zigzag and armchair directions). Silicon and carbon monochalcogenides present a broad range of values for the effective masses. In this respect, SiTe and SiS have particularly interesting properties. While the anisotropy is less evident than in phosphorene, SiTe presents significantly small effective masses hinting to a high mobility in both the armchair and zigzag directions. This property might be useful to realize transistors [8,12]. In a different way, the SiS monolayer presents hole effective masses strongly dependent on the direction, with a value in the zigzag direction almost 100 times larger than the value in the armchair direction. Accordingly, this material is expected to exhibit a huge anisotropy in the hole mobility that might be of interest for less conventional plasmonic and thermoelectric devices, as discussed, for example, in [12].

## Conclusion

In this work we applied ab initio techniques to study the properties of recently proposed two-dimensional compounds with the structure of phosphorene. Specifically, we considered carbon and silicon monochalcogenides and found the CS, CSe, CTe, SiO, SiS, SiSe, and SiTe monolayers to be dynamically stable. Then, the electronic properties of these new materials were determined, and the results indicate that these compounds span a broad range of electronic bandgaps and charge carrier mobilities, showing potential for future technological applications.
